# Novel compound heterozygous mutation in *STAMBP* causes a neurodevelopmental disorder by disrupting cortical proliferation

**DOI:** 10.3389/fnins.2022.963813

**Published:** 2022-08-10

**Authors:** Meixin Hu, Huiping Li, Zhuxi Huang, Dongyun Li, Ying Xu, Qiong Xu, Bo Chen, Yi Wang, Jingxin Deng, Ming Zhu, Weijun Feng, Xiu Xu

**Affiliations:** ^1^Department of Child Health Care, Children’s Hospital of Fudan University, National Children’s Medical Center, Shanghai, China; ^2^Institute of Pediatrics, Children’s Hospital of Fudan University, Shanghai, China; ^3^Shanghai Key Laboratory of Medical Epigenetics, International Co-Laboratory of Medical Epigenetics and Metabolism, Institutes of Biomedical Sciences, Shanghai Medical College, Fudan University, Shanghai, China

**Keywords:** *STAMBP*, neurodevelopmental disorder, microcephaly, cortical organoids, novel mutation

## Abstract

**Background:**

Mutations in the *STAMBP* gene, which encodes a deubiquitinating isopeptidase called STAM-binding protein, are related to global developmental delay, microcephaly, and capillary malformation. Owing to the limited number of reported cases, the functional and phenotypic characteristics of *STAMBP* variants require further elucidation.

**Materials and methods:**

Whole exome sequencing was performed on a patient presenting with a neurodevelopmental disorder. Novel compound heterozygous mutations in *STAMBP* [c.843_844del (p.C282Wfs*11) and c.920G > A (p.G307E)] were identified and validated using Sanger sequencing. A 3D human cortical organoid model was used to investigate the function of *STAMBP* and the pathogenicity of the novel mutation (c.920G > A, p.G307E).

**Results:**

The patient was presented with global developmental delay, autism spectrum disorder, microcephaly, epilepsy, and dysmorphic facial features but without apparent capillary malformation on the skin and organs. Cortical organoids with *STAMBP* knockout (KO) showed significantly lower proliferation of neural stem cells (NSCs), leading to smaller organoids that are characteristic of microcephaly. Furthermore, *STAMBP* disruption did not affect apoptosis in early cortical organoids. After re-expressing wild-type STAMBP, STAMBP^*G*307*E*^, and STAMBP^*T*313*I*^ (a known pathogenic mutation) within *STAMBP* KO organoids, only STAMBP^*WT*^ rescued the impaired proliferation of *STAMBP* deficient organoids, but not STAMBP^*G*307*E*^ and STAMBP^*T*313*I*^.

**Conclusion:**

Our findings demonstrate that the clinical phenotype of *STAMBP* mutations is highly variable, and patients with different *STAMBP* mutations show differences in the severity of symptoms. The *STAMBP* missense mutation identified here is a novel pathogenic mutation that impairs the proliferation of NSCs in human brain development.

## Introduction

The *STAMBP* gene (alias *AMSH*, associated molecule with the SH3 domain of STAM) on chromosome 2p13 encodes a deubiquitinating (DUB) isopeptidase called STAM-binding protein (Tanaka et al., 1999). Homozygous or compound heterozygous mutations in *STAMBP* have been reported to be pathogenic. Patients with microcephaly-capillary malformation syndrome (MICCAP: OMIM #614261) have biallelic mutations in the *STAMBP* gene (McDonell et al., 2013; Pavlović et al., 2014; Faqeih et al., 2015; Naseer et al., 2016; Demikova et al., 2018; Hori et al., 2018; Lm, 2019; Wu et al., 2019). The phenotype of MICCAP consists of global developmental delay, progressive microcephaly, intractable epilepsy, and generalized capillary malformations on the skin. However, the number of reported cases with *STAMBP* mutations is limited. The functional and phenotypic characteristics of mutations in *STAMBP* have not been fully elucidated.

The STAMBP protein contains a microtubule-interacting and transport (MIT) domain and a STAM-binding motif, both of which interact with endosomal sorting and trafficking machinery (Agromayor and Martin-Serrano, 2006; Wright et al., 2011; Davies et al., 2013). STAMBP is important for cell surface receptor-mediated endocytosis and sorting. Previously, it was reported that insensitive activation of the RAS-mitogen activated protein kinase (RAS-MAPK) and PI3K-AKT-mTOR pathways might contribute to vascular and capillary malformation and apoptosis induction by a defective DUB, which may be responsible for microcephaly (McDonell et al., 2013). Interestingly, one study failed to repeat the constitutive activation of the PI3K-AKT-mTOR pathway in patient-derived lymphoblastoid cell lines (LCLs) with a novel *STAMBP* mutation (Hori et al., 2018). This raises the question of whether different mutations in *STAMBP* affect different signaling pathways and lead to a variable clinical phenotype.

Studies involving the *Stambp*^–/–^ mice found that homozygous knockout mice were morphologically indistinguishable from their littermates at birth, and histopathological examination revealed normal morphogenesis in all tissues tested (Ishii et al., 2001). Early postnatal mice display neurodegenerative apoptotic activation and ubiquitin-conjugated protein aggregation in the hippocampus and cerebral cortex (Suzuki et al., 2011). However, patients carrying *STAMBP* mutations exhibited clinical phenotypes congenitally, indicating that the function of *STAMBP* in humans is different from mice. To address the gap between mouse models and human diseases, human brain organoids generated from pluripotent stem cells have emerged as a promising approach for investigating the disease characteristics in relevant cellular and genetic contexts (Lancaster et al., 2013; Chen et al., 2014; Bershteyn et al., 2017; Quadrato et al., 2017).

Here, in this study, we present a Chinese patient diagnosed with a neurodevelopmental disorder carrying compound heterozygous *STAMBP* mutations, including a novel STAMBP^*G*307*E*^ mutation. Using the human cortical organoids (hCOs) model, we showed that the *STAMBP* deficiency disrupts the proliferation of neural stem cells (NSCs), leading to a dramatic size reduction in hCOs. We also found that the newly identified STAMBP^*G*307*E*^ mutation could not rescue phenotypes caused by *STAMBP* deficiency.

## Patient and methods

### Ethics statement

Ethical approval for the present study was obtained from the Ethics Committee of Children’s Hospital affiliated with Fudan University (permit no. 2016-131). Informed consent was obtained from the patient’s parents.

### Patient

The patient who was a girl 3 years and 11 months old was the only child of healthy non-consanguineous Chinese parents (pregnant in their early 30s). She was delivered at term *via* normal spontaneous vaginal delivery, with a birth weight of 2800 g (15th percentile, according to the WHO Child Growth Standards). Birth length and head circumference have not been reported.

Physical examination revealed an apparent growth delay, especially in head circumference. At 18 months of age, her weight was 8.6 kg (5th–15th percentile), height was 75.1 cm (1st–3rd percentile), and head circumference was 42.5 cm (<1st percentile). At 3.5 years, her weight was 12.1 kg (5th percentile), her height was 85.1 cm (<1st percentile), and her head circumference was 44.8 cm (<1st percentile). Dysmorphic features included hypertelorism, low-set ears, anteverted nares, and drooping corners of the mouth. In contrast to the previously reported cases, the girl did not show apparent capillary malformations on the skin. Ultrasound examination revealed no capillary malformation in the liver, spleen, or kidneys.

**FIGURE 1:**
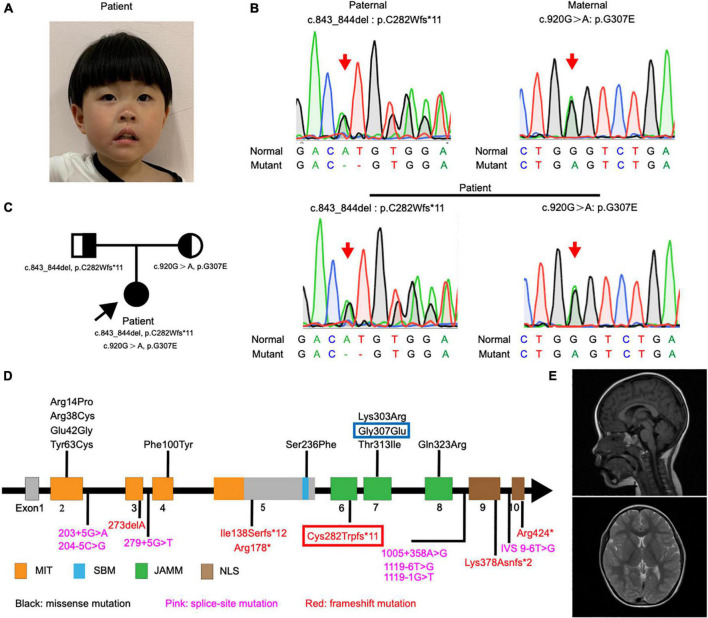
Detection and location of *STAMBP* mutation in case patient. **(A)** Clinical photograph showing craniofacial phenotypes (hypertelorism, wide nasal bridge, anteverted nares, and drooping corners of the mouth). **(B)** Sanger sequencing of the *STAMBP* mutations showed that the case patient has compound heterozygous mutations from her father (c.843_844del, p. C282Wfs*11) and mother (c.920G > A, p.G307E) (arrow). **(C)** Pedigree of the patient. **(D)** Diagram indicates the location of the patients’ mutations of *STAMBP* described in previous reports and ours. Blue frame labels this case’s mutation inherited from her mother. Red frame labels this case’s mutation inherited from her father. STAMBP contains a microtubule-interacting and transport (MIT) domain, an SH3 binding motif (SBM), a JAMM (JAB/MPN/MOV34) motif, and a nuclear localization signal (NLS). **(E)** Representative images showing cerebral MRI scans of the patient.

Her seizures began in infancy and manifested as apnea and eyes turning up during sleep (generally within 10–20 min of falling asleep) without limb shaking or stiffness and restored consciousness after several minutes. Even with a small head circumference, cerebral magnetic resonance imaging (MRI) with spectroscopy revealed no remarkable anomalies (Figure 1E). However, the electroencephalogram (EEG) examination was strikingly abnormal, with generalized epileptogenic activity.

She had a global developmental delay and did not walk until 20 months of age. Her speech and language acquisition were delayed and she only could say “mama” and “papa.” She avoided eye contact and did not respond to the adults’ simple instructions. She was hyperactive and exhibited a series of repetitive hand movements, such as putting her hands in front of her eyes, flicking her fingers, digging her fingers into a wall or book, and flipping through books.

She has suffered from feeding problems, refused to accept new foods, and rejected drinking milk and eating fruits and vegetables. Her major food sources were limited to rice and eggs. We performed a laboratory examination of liver and kidney function. Tests showed that she had hyperuricemia and hypercholesterolemia (blood uric acid was 522 μmol/L; total cholesterol was 6.39 mmol/L; high-density lipoprotein was 2.07 mmol/L; free fatty acid was 634 μmol/L). After 2 months of dietary adjustment, her blood tests of uric acid and lipid levels were recovered (blood uric acid was 348 μmol/L; total cholesterol was 5.02 mmol/L; high-density lipoprotein was 1.66 mmol/L; free fatty acid was 794 μmol/L).

### Variation analysis

Peripheral blood samples (2 ml) taken from the patient and her parents were collected into tubes containing EDTA anticoagulant. Sample preparation and whole-exome sequencing were performed at the Molecular Genetic Diagnosis Center of Children’s Hospital of Fudan University. The genomic DNA of all samples was sequenced using the HiSeq 2000 platform (Illumina). Sequencing data were generated and assembled using the Ensemble GRCh37/hg19 reference genome. Mutation validation was performed using Sanger sequencing with two primer pairs to amplify exon 6 (chr2:74076590-74076591, forward primer:5′-AGGGCTCAGTGGTCGCAGA-3′; reverse primer:5′-GAGAG TCACAGGATGCCAAGAG-3′) and exon 7 (chr2:74077555, forward primer:5′-GCTTACCTTTCCACTGTCGG-3′; reverse primer,5′-TAAAAGCCCTAAGTGTTCCCAGA-3′) of the *STAMBP* gene (NM_006463). Mutations were predicted using Addgene software. The pathogenicity of the genetic variants was predicted using bioinformatics tools, such as PolyPhen-2,^1^ PROVEAN, and SIFT.^2^ The variants were classified according to American College of Medical Genetics and Genomics (ACMG) guidelines.

### Literature review

The PubMed,^3^ CNKI,^4^ Wanfang,^5^ and ClinVar^6^ databases were used to retrieve previous studies with the keywords of “STAMBP or AMSH” until April 2022.

### Human embryonic stem cells culture

H9 human embryonic stem cells (hESCs) were cultured on Matrigel-coated tissue culture dishes containing mTeSR plus medium. Cells were passaged every 4 days using EDTA (0.5 mM).

### CRISPR/Cas9-based genome editing

The CRISPR guide for *STAMBP* was designed using the Benchling CRISPR Guide Design Tool.^7^ The guide was designed to maximize on-target efficiency and minimize off-target sites in the intragenic regions. *STAMBP* sgRNA:5′-TCCCAAAGCAGAAGAGCTGA -3′ (PAM: AGG). The corresponding coding sequence was cloned into the pSpCas9(BB)-2A-Puro (PX459) construct (Addgene, plasmid #62988). A total of 1 × 10^6^ of single cells dissociated from H9 hESCs cultured were transfected with 3 μg PX459-sgRNA using Lipofectamine 3000. After transfection, the cells were plated onto Matrigel-coated 6-well plates with mTeSR plus medium containing 10 μM Y27632. Two days of puromycin selection were performed starting 24 h after transfection. After 5–7 days of recovery, the cells were dissociated into single cells using Accutase and cultured in Matrigel-coated 96-well plates. After 10–14 days of culture, half of each clone was collected for genomic DNA isolation. Clones were screened using PCR and Sanger sequencing. The target clones were expanded for downstream use.

### Cortical organoids generation

Human cortical organoids (hCOs) were generated using a protocol adapted from the study done by Xiang et al. (2017). H9 hESCs were dissociated using Accutase and cultured in neural induction medium (DMEM-F12, 15% (v/v) KSR, 1% (v/v) MEM-NEAA, 1% (v/v) GlutaMAXmax, 100 μM β-Mercaptoethanol, 100 nM LDN-193189, 10 nM SB-431542, and 2 μM XAV-939) supplemented with 50 μM Y27632 and 5% (v/v) FBS. A total of 9,000 cells were plated in each well of an ultra-low-attachment 96-well plate. Neural induction media were replenished every other day until day 10 (FBS was removed from day 2 and Y27632 was removed from day 4). On day 10, organoids were transferred into ultra-low-attachment 6-well plates for spinning culture (85 rpm) and cultured with neuronal differentiation media (1:1 mixture DMEM-F12 and Neurobasal media, 0.5% (v/v) N2 supplement, 1% (v/v) B27 supplement without vitamin A, 1% (v/v) Glutamax, 0.5% (v/v) MEM-NEAA, 0.025% (v/v) insulin, 50 μM β-Mercaptoethanol, and 1% (v/v) penicillin/streptomycin). Neural differential media were replenished every other day until day 18. On day 18, a B27 supplement with vitamin A was used, and 20 ng/ml BDNF, 200 μM ascorbic acid, and 200 μM cAMP were added. The medium was replenished every 3 days thereafter.

### Western blot analysis

Human embryonic stem cells were lysed in a buffer containing 20 mM Tris-HCI (pH 8.0), 1 mM EDTA, 1 mM EGTA, 1% Triton X-100, 450 mM NaCl, 1 × protease inhibitor cocktail (Roche). Protein samples were loaded onto a 10% PAGE gel (Epizyme) and transferred onto PVDF membranes. The membranes then were incubated with anti-SATMBP (mouse, SCBT sc-271641, 1:1,000), anti-Flag (mouse, Shanghai Genomics GNI4110-FG, 1:1000) at 4°C overnight and incubated with secondary antibodies at room temperature for 1 h. The blots were developed using SuperSignal West Femto Maximum Sensitivity Substrate (Thermo Fisher Scientific) and the ChemiDoc System (Bio-Rad).

### RT–qPCR

All RNA samples were extracted using the TRIzol reagent (Sigma-Aldrich). The PrimeScript RT Master Mix (Takara) was used for cDNA synthesis. Quantitative PCR was performed using TB Green Premix Ex Taq II (Takara) on a CFX384 Touch Real-Time PCR Detection System (Bio-Rad). The primers used for RT-qPCR are listed in the Supplementary Table 3.

### Immunostaining and fluorescence quantification

Organoids were fixed in 4% paraformaldehyde for 2 h at room temperature, followed by washing with PBS 3 times for 5 min each. The fixed organoids were then allowed to sink in 30% sucrose overnight at 4°C, then embedded in O.C.T. and cryosectioned at 20 μm. The organoid sections were blocked with 3% donkey serum, 0.2% TritonX-100, and 0.1% Tween in PBS for 1 h at RT. Then, the samples were incubated with primary antibodies overnight at 4°C and secondary antibodies for 1 h at RT. Images of these sections were taken using a confocal microscope (Leica TSC SP8). ImageJ software was used for image processing and quantification of fluorescence intensity. These methods have been previously described (Guo et al., 2021). To measure the surface area of the organoid, a circle was drawn surrounding it and quantified using ImageJ software. To measure the areas of SOX2-positive cells, a circle was drawn surrounding the ventricular zone (VZ)-like a rosette and quantified using the ImageJ software. The mean fluorescence signal in each section was detected and quantified using ImageJ to measure the fluorescence intensity of Ki67-positive, PH3-positive, and CC3-positive cells. The antibodies used for immunostaining are listed in the Supplementary Table 2.

### *STAMBP* knockout rescue experiment

FLAG-HA-STAMBP plasmid was purchased from Addgene (#22560). The FLAG-tagged STAMBP^*WT*^, FLAG-tagged STAMBP^*G*307*E*^, and FLAG-tagged STAMBP^*T*313*I*^ sequences were cloned into the PPB-FH-IRES-PuroR vector. For STAMBP overexpression-mediated rescue experiments, *STAMBP* KO hESCs were overexpressing FLAG-tagged STAMBP^*WT*^, FLAG-tagged STAMBP^*G*307*E*^, or FLAG-tagged STAMBP^*T*313*I*^ by plasmid transfection using Lipofectamine 3000 followed by the generation of cortical organoids.

### Statistical analysis

All the raw data are showed in the Supplementary Data Sheet 1. The data were analyzed and visualized using GraphPad PRISM 7.0a. The ShapiroWilk test and the Kolmogorov–Smirnov test were used to test for normality. Normally distributed datasets were compared using Student’s *t*-test and one-way ANOVA with Dunnett’s multiple comparison tests. Values are presented as mean ± SEM. Data that were not normally distributed were analyzed using the Mann–Whitney test and Kruskal–Wallis ANOVA with Dunn’s multiple comparisons tests and presented as a median ± 95% confidence interval. All statistical tests were two-tailed, and statistical significance was defined as *p* < 0.05. *p < 0.05, ^**^p < 0.01, ^***^p < 0.001.

## Results

### The clinical features and molecular phenotype of the patient

The patient was diagnosed with global developmental delay and autism spectrum disorder at 3 years of age. Consistent with the clinical observations, cognitive/developmental evaluation using the Griffiths Mental Development Scales (GMDS) showed that her locomotor, personal-social, hearing-speech, coordination, and performance skills were well below the age range (<1st percentile). The Autism Diagnostic Observation Schedule, second edition (ADOS-2) Module 1 was used to assess autism spectrum disorders (ASD). The results of the ADOS-2 (score of social effect: 20; restricted and repetitive behavior: 5; overall total score: 25) led to the diagnosis of autism with a high level of symptomatology.

The patient showed a short stature and microcephaly. She also showed dysmorphic features including hypertelorism, low-set ears, and drooping corners of the mouth. Notably, we also observed a new feature of anteverted nares (Figure 1A).

Exome sequencing of genomic DNA from the patient revealed the presence of compound heterozygous mutations c.843_844del (p.C282Wfs*11) and c.920G > A (p.G307E) in the S*TAMBP* gene. Her father and mother were heterozygous for c.843_844del and c.920G > A, respectively. Sanger sequencing was performed to validate the mutations (Figure 1B). The mode of inheritance in the proband is consistent with an autosomal recessive pattern, shown by pedigree analysis (Figure 1C). After reviewing *STAMBP* variants in patients reported previously, we summarized phenotypes and mutations in *STAMBP*: 10 missense mutations, six different frameshift mutations predicted to cause premature truncation of the STAMBP protein, and seven intronic mutations leading to alternative splicing of the *STAMBP* transcript (Figure 1D).

Most patients with *STAMBP* mutations have shown global developmental delay, microcephaly, capillary malformation, epilepsy, and dysmorphic features. Our patient met most of the characteristics of mutation in *STAMBP* (Table 1 and Supplementary Table 1). Unlike previously reported cases, this girl did not show any obvious capillary malformations on the skin.

**TABLE 1:** Summary of *STAMBP* mutant patients’ early development and evaluation results.

Subjects	This case	Reported cases
Gender	Female	15/20, male
Age	3 years 11 months	22 days–12 years
Short stature	Yes	15/20
Microcephaly	Yes	20/20
Epilepsy	Yes	20/20
Developmental delay	Yes	20/20
Autism-like behavior	Yes	2 (18 NA)/20
Age of walking (months)	20	—
Age of speaking (months)	Non-verbal at evaluation	Non-verbal at evaluation, 4 (16 NA)/20
Comorbidity		
Feeding problem	Yes	2 (18 NA)/20
Sleep disorder	No	2 (18 NA)/20
Clinical evaluations		
Age of GMDS (months)	35	—
Locomotor	<1st percentile	
Personal-social	<1st percentile	
Hearing-speech	<1st percentile	
Hand-eye coordination	<1st percentile	
Performance	<1st percentile	
Age of ADOS (months)	35	—
Social affect	20	
Restricted and repetitive behavior	5	
Total score	25 (ASD cut-off: 11)	

NA, not available; GMDS, Griffiths Mental Development Scales; ADOS, Autism Diagnostic Observation Schedule, second edition.

### Protein consequences of *STAMBP* mutation

**FIGURE 2:**
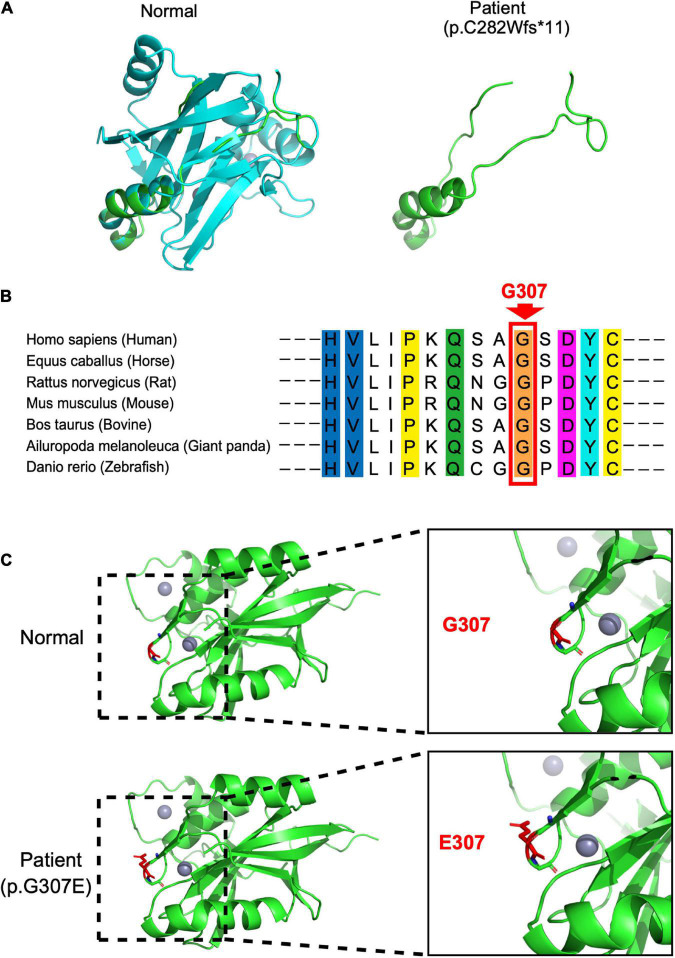
Protein consequences of *STAMBP* mutation. **(A)** Superimposition of the STAMBP (p.C283Wfs*11) protein was achieved using SWISS-MODEL and visualized using PyMOL. The protein structures of STAMBP (left) and STAMBP (p.C283Wfs*11) variation (right). The truncated protein is shown in green. **(B)** Conservation of mutated residue across evolution. **(C)** Superimposition of STAMBP (p.G307E) protein was performed using SWISS-MODEL and visualized using PyMOL. Expanded view of patient mutation (p.G307E) showing alternation of the Hydrogen bonds (red dashes) in a loop structure. Also, gray spheres show two bound Zn^2+^ atoms. α-helix and β-sheet are shown in green.

In our case, the patient carried compound heterozygous paternal (c. 843_844del, p. C282Wfs * 11) and maternal (c. 920G > A, p. G307E) mutations. After searching the PubMed, CNKI, Wanfang, and ClinVar databases as of April 2022, we found that p.C282Wfs*11 has been reported to be pathogenic in ClinVar (Variation ID:1034283), while p.G307E has not been reported. The protein structure of STAMBP (p.C282Wfs*11), predicted using SWISS-MODEL, showed a truncated protein caused by a frameshift mutation with a 2-basepair (AT) deletion (Figure 2A). For the protein consequences of STAMBP (p.G307E) mutation, multiple sequence alignment (MSA) showed that the missense mutation (c.920G > A) converts a conserved glycine residue to glutamic acid (p.G307E) (Figure 2B). The functional prediction programs PolyPhen-2, PROVEAN, and SIFT (Kumar et al., 2009; Adzhubei et al., 2010; Choi and Chan, 2015) were used to determine the effects of the p. G307E mutation. PolyPhen-2 predicted the change to be “possibly damaging” with a score of “0.763”; PROVEAN predicted the mutation to be “deleterious” with a score of “−3.25” and SIFT predicted the mutation to be “damaging” with a score of “0.010.” In addition, the protein structure of the mutation p.G307E was predicted using SWISS-MODEL and PyMOL (Schwede et al., 2003; Johansson et al., 2012). The mutation p.G307E was located at the loop structure within the JAMM domain, which has the function of binding with ubiquitinated proteins. Altering hydrogen bonds at the side chain position was predicted to affect the function of the STAMBP protein (Figure 2C). Taken together, these predictions suggest that p. G307E likely has a negative impact on STAMBP function.

### STAMBP is expressed in the developing human brain

**FIGURE 3:**
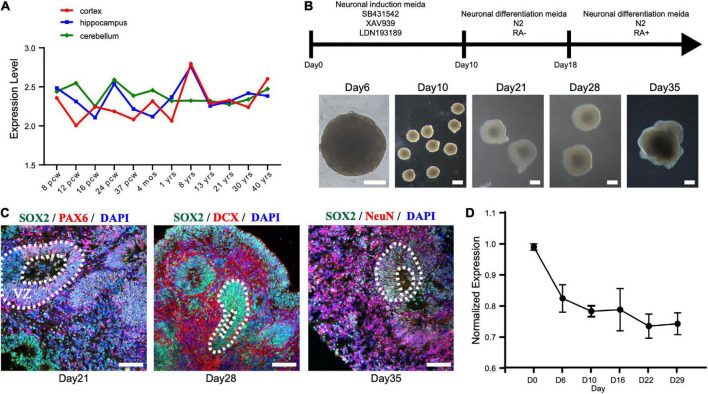
STAMBP expression during human cortical development. **(A)** STAMBP expression in the human cortex, hippocampus, and cerebellum. Data were downloaded from Allen Brain Atlas (available from: http://www.brain-map.org; Ensemble ID: ENSG00000124356). pcw, post-conceptional weeks; mos, months; yrs, years. **(B)** Schematic representation of the culture system described in detail in Section “Patient and methods.” Examples of each stage are shown. Scale bars, 200 μm. **(C)** Immunofluorescent staining of hCOs of day 21 for neural stem cell markers Left column: SOX2, PAX6, and DAPI; middle column: SOX2, DCX, and DAPI; right column: SOX2, NeuN, and DAPI. Scale bars, 100 μm. **(D)** *STAMBP* mRNA expression in the different differentiated stages of hCOs. Mean ± SEM from three independent experiments.

First, we investigated the expression pattern of *STAMBP* in the human brain. Using the public gene-expression database, the Allen Brain Atlas, which characterizes the spatiotemporal expression pattern of the human brain, the data showed that *STAMBP* was expressed 8 weeks after conception and stably expressed into adulthood. *STAMBP* was widely expressed in multiple brain areas, including the cerebral cortex, hippocampus, and cerebellum (Figure 3A).

To test the expression of *STAMBP* during human brain development, we generated hESCs derived hCOs using H9 hESCs (Figure 3B). We found that radially organized cells resembling the VZ were enriched with SOX2^+^ and PAX6^+^ cells on day 21, confirming their identity as NSCs (Figure 3C, left column). The neuroblast-specific marker, doublecortin (DCX), was expressed on the outer side of SOX2^+^ NSCs on day 28 (Figure 3C, middle column). On day 35, NeuN, indicative of differentiated neurons, was observed outside the VZ-like areas (Figure 3C, right column). These results demonstrate that hCOs differentiated to a similar extent and acquired morphological features of organized cortical structures. RT-PCR was performed to analyze the expression patterns of *STAMBP* mRNA at different developmental stages. The results showed that *STAMBP* was expressed in hESCs and its expression was maintained during the induction and differentiation stages of cortical organoids (Figure 3D).

### *STAMBP* deletion results in smaller cortical organoids and impairs neural stem cell proliferation

**FIGURE 4:**
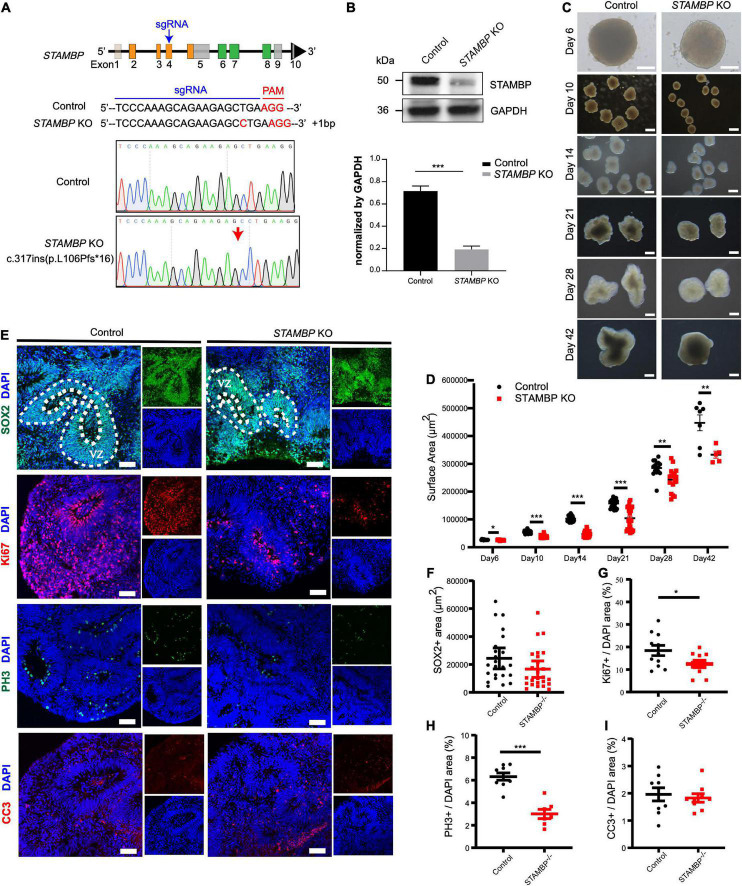
*STAMBP* deletion results in smaller cortical organoids sizes and impairs NSC proliferation. **(A)** CRISPR/Cas9-mediated gene editing of human *STAMBP* locus in hESCs resulted in a 1 bp insertion in exon 4. **(B)** Western blot showed ablation of STAMBP proteins due to premature mature stop codons in *STAMBP* KO hESCs. **(C)** Representative images of control and *STAMBP* KO cortical organoids at different differential stages. Scale bars, 200 μm. **(D)** Quantification of surface areas of cortical organoids in different developmental stages. Each plot represented an individual organoid. Experiments were repeated three times. Data of day 6, day 21, day 28, and day 42 organoids are presented as mean ± SEM with student’s *t*-tests. Data of day 10 and day 14 organoids are presented as a median ± 95% confidence interval with the Mann–Whitney test. *p < 0.05, **p < 0.01, ***p < 0.001. **(E)** Immunofluorescent staining was performed on sections of control and *STAMBP* KO cortical organoids on day 28. Sections were stained with SOX2 and DAPI (the first row), Ki67 and DAPI (the second row), PH3 and DAPI (the third row), and CC3 and DAPI (the fourth row). Scale bar: 100 μm. **(F–I)** Quantification of the VZ-like SOX2^+^ rosette area **(F)**, the ratio of Ki67^+^ vs. DAPI **(G)**, the ratio of PH3^+^ vs. DAPI **(H)**, and the ratio of CC3^+^ vs. DAPI **(I)**. Experiments were repeated three times. Data of SOX2^+^ area are presented as median ± 95% confidence interval with the Mann–Whitney test. Data of Ki67^+^/DAPI, PH3^+^/DAPI, and CC3^+^/DAPI are presented as mean ± SEM with student’s *t*-tests. *p < 0.05, ***p < 0.001.

Next, we generated *STAMBP* mutant hESCs using the CRISPR/Cas9 approach (Ran et al., 2013). *STAMBP* KO hESCs occurred as one cytosine nucleotide insertion in exon 4, resulting in a frameshift, leading to the generation of a premature stop codon (Figure 4A). Western blotting confirmed the absence of STAMBP in the mutant hESCs (Figure 4B). To examine whether the deletion of *STAMBP* affects the pluripotency of hESCs, we examined the expression of pluripotent markers. Immunostaining and RT-PCR were performed to determine the expression of pluripotent markers, including SOX2, NANOG, and REX1. The results showed no significant changes in the expression of pluripotency markers, suggesting *STAMBP* deletion did not affect the pluripotency of hESCs (Supplementary Figures 1A,B).

The size of organoids generated from *STAMBP* KO hESCs and wild-type H9 cells was measured at different time points. *STAMBP* KO hCOs were drastically smaller in size and showed significantly reduced surface areas compared to wild-type H9 hCOs from day 10 (Figures 4C,D). Microcephaly may be caused by the abnormal proliferation and apoptosis of NSCs (Thornton and Woods, 2009; Manzini and Walsh, 2011). Therefore, we immunostained the NSC marker SOX2 to indicate VZ-like regions on day 28 organoids and measured the area of SOX2^+^ VZ-like regions. Regardless of not reaching the threshold for statistical significance, *STAMBP* KO organoids showed a trend of reduction in SOX2^+^ VZ-like regions (Figures 4E, first row,F). The proliferation of *STAMBP* KO organoids was monitored by immunostaining for the cell cycle marker Ki67, and the results showed a decrease in cell proliferation in *STAMBP* KO organoids compared to control organoids (Figures 4E second row,G). We also stained for the mitotic cell marker phospho-histone 3 (PH3) on day 28 organoids. The results showed a significantly reduced PH3^+^/DAPI area in *STAMBP* KO organoids as compared to controls (Figures 4E third row,H), indicating impaired proliferation of NSC in *STAMBP*-deficient hCOs. Given that previous studies in a mouse model showed that loss of STAMBP causes apoptosis in neuronal cells, we also stained for the apoptosis marker cleaved caspase 3 (CC3) (Figure 4E, fourth row). In contrast to the findings in the mouse model, our data showed no difference in apoptosis between *STAMBP* KO hCOs and wild-type H9 hCOs (Figure 4I). Collectively, these results suggest a disruption of NSC proliferation in *STAMBP* mutant organoids.

### Overexpression of the patient’s missense mutation, STAMBP^*G*307*E*^, could not rescue the impaired proliferation of neural stem cells in *STAMBP* deficient organoids

**FIGURE 5:**
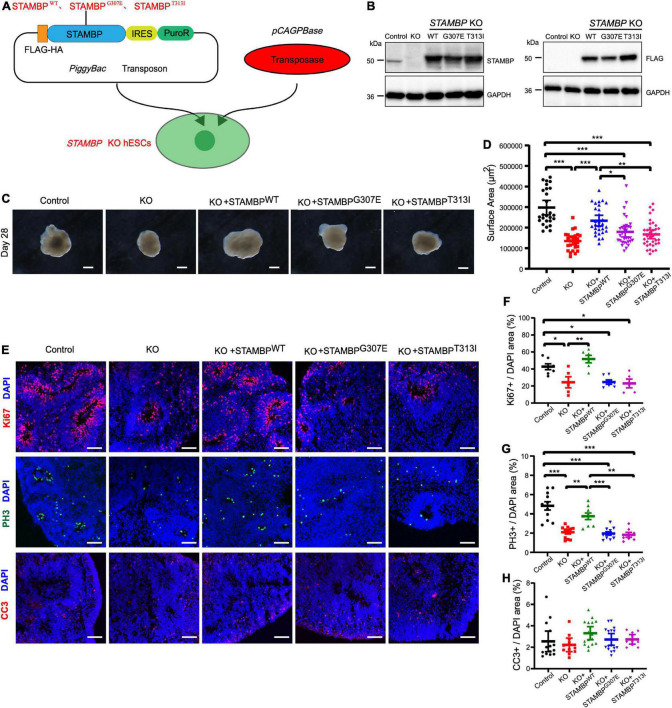
STAMBP^*WT*^ but not STAMBP^*G307E*^ and STAMBP^*T313I*^ overexpression rescues the phenotypes of reduced cortical size and impaired proliferation of NSCs. **(A)** Schematic representation of the PiggyBac-mediated STAMBP expression system. **(B)** Western blotting confirmed the presence of STAMBP^*WT*^, STAMBP^*G307E*^, and STAMBP^*T313I*^ protein generated from the *STAMBP* KO hESCs. **(C)** Representative images of control, *STAMBP* KO cortical organoids, and organoids generated from STAMBP^*WT*^, STAMBP^*G307E*^, and STAMBP^*T313I*^ overexpression on day 28. Scale bars, 200 μm. **(D)** Quantification of surface areas of cortical organoids. Each plot represented an individual organoid. Experiments were repeated three times. Groups were compared using a Kruskal–Wallis ANOVA with Dunn’s multiple comparisons tests. Data are presented as median ± 95% confidence interval, *p < 0.05, **p < 0.01, ***p < 0.001. **(E)** Immunofluorescent staining was performed on organoids on day 28. Sections were stained with Ki67 (the first row), PH3 (the second row), and CC3 (the third row). Scale bar: 100 μm. **(F–H)** Quantification of cell cycling marker Ki67-positive cells **(F)**, PH3-positive cells **(G)**, and cell apoptosis marker CC3 **(H)**. Each plot represented an individual section. Experiments were repeated three times. Data of Ki67^+^/DAPI and PH3^+^/DAPI were compared using a one-way ANOVA followed by a Dunnett’s multiple comparisons test. Data are shown as mean ± SEM. Data of CC3^+^/DAPI were using a Kruskal–Wallis ANOVA with Dunn’s multiple comparisons tests. Data are presented as a median ± 95% confidence interval. *p < 0.05, **p < 0.01, ***p < 0.001.

To confirm the causal relationship between *STAMBP* deletion and phenotypes of cortical organoids, we used the PiggyBac transposon system to transpose the *STAMBP* gene into *STAMBP* KO hESCs (Figure 5A). Furthermore, it was unknown whether the novel missense variant STAMBP^*G*307*E*^ was pathogenic in this patient. Hence, we overexpressed FLAG-STAMBP^*WT*^, FLAG-STAMBP^*G*307*E*^, and FLAG-STAMBP^*T*313*I*^, a previously reported *STAMBP* missense variant (c.938C > T, Thr313Ile) in *STAMBP* KO hESCs (Supplementary Figure 2A). The missense mutations were confirmed with Sanger sequencing (Supplementary Figure 2B). Western blotting confirmed the re-expression of STAMBP in *STAMBP* deficient hESCs (Figure 5B).

We subsequently repeated the experiments following the same organoid differentiation protocol as described above. Overexpressed STAMBP^*WT*^ in *STAMBP* KO hESCs increased the generated organoids’ size compared to *STAMBP* KO organoids on day 28, whereas overexpressed STAMBP^*G*307*E*^ and STAMBP^*T*313*I*^ failed to rescue the size of *STAMBP* deficient organoids (Figures 5C,D).

In addition, we examined whether impaired cellular proliferation in *STAMBP* KO organoids could be rescued. Immunostaining analysis showed that the decreased Ki67^+^ and PH3^+^ NSCs pools were partially rescued in organoids generated from STAMBP^*WT*^ overexpression. However, organoids generated from STAMBP^*G*307*E*^ and STAMBP^*T*313*I*^ overexpression showed an indistinguishable difference in Ki67^+^ and PH3^+^ cells compared to *STAMBP* KO organoids on day 28 (Figures 5E first and second rows,F,G). To further analyze the possible impact on apoptosis, we also stained the apoptosis marker, CC3, in organoids generated from the STAMBP^*G*307*E*^ and STAMBP^*T*313*I*^ overexpressing hESCs. The results showed that there was no significant difference in the CC3^+^/DAPI areas of hCOs generated from STAMBP^*G*307*E*^, STAMBP^*T*313*I*^, and STAMBP^*WT*^ compared to control or *STAMBP* KO hCOs on day 28 (Figures 5E third row,H). Together, these results demonstrate that the decreased size of *STAMBP* deficient cortical organoids is mainly due to the impaired proliferation of NSCs. The *STAMBP* missense variant, STAMBP^*G*307*E*^ is a novel pathogenic variant. The impaired proliferation of NSCs may be a major contributor to the pathogenic mechanism in our patients.

## Discussion

In this study, we describe a girl with global developmental delay, autism spectrum disorder, microcephaly, and minor dysmorphic facial features. She had compound heterozygous mutations of *STAMBP* [c.843_844del (p.C282Wfs*11) and c.920G > A (p.G307E)] inherited from her parents. MSA, PolyPhen-2, PROVEAN, and SIFT software were used to predict the pathogenicity of the novel mutation (c.920G > A, p.G307E). These predictions suggested that p.G307E is likely to have a negative impact on STAMBP function. We also used human cortical organoids to investigate the effects of this novel mutation. The data showed that in contrast to STAMBP^*WT*^, STAMBP^*G*307*E*^ overexpression could not rescue the impaired proliferation of NSCs in *STAMBP*-deficient organoids. We suggest that the missense mutation (c.920G > A) in the *STAMBP* gene is a novel pathogenic mutation.

At least 20 cases identified with *STAMBP* gene mutation have been reported (McDonell et al., 2013; Pavlović et al., 2014; Faqeih et al., 2015; Naseer et al., 2016; Demikova et al., 2018; Hori et al., 2018; Lm, 2019; Wu et al., 2019). Global developmental delay, progressive microcephaly, epilepsy, and capillary malformations are the typical clinical manifestations. The patient described here showed most of these symptoms. However, no capillary malformations were found on her skin, and it was difficult to find such malformations on the internal organs by ultrasound. Compared with those of previously reported patients, this patient’s symptoms were relatively mild. Capillary malformations are cutaneous vascular abnormalities associated with RAS-MAPK pathway dysregulation (Tidyman and Rauen, 2009). The pathogenesis of capillary malformation in *STAMBP* mutations has not been completely addressed. McDonell et al. (2013) found that STAMBP interacts with the Grb2 adaptor, an important component of the RAS signal transduction pathway, and they proposed that the RAS pathway may be responsible for capillary abnormalities in patients with *STAMBP* mutation. Different mutation sites of *STAMBP* may have different downstream targets and cause various symptoms, which is supported by a study of the Ser236Phe mutation of *STAMBP* (McDonell et al., 2013; Hori et al., 2018). Therefore, we propose that capillary malformation is not a definite requirement for *STAMBP* mutation-related disease. However, we need to follow up with the patient and observe the changes in her clinical features.

Human brain size is determined through a tightly orchestrated and intricate process of neural stem cell proliferation, migration, organization, apoptosis, and cell growth (Pirozzi et al., 2018). The delicate study by McDonell et al. (2013) showed that reduced STAMBP expression was associated with the accumulation of ubiquitin-conjugated protein aggregates, elevated apoptosis, and insensitive activation of the RAS-MAPK and PI3K-AKT-mTOR pathways in lymphoblastoid cell lines (LCLs) from patients with *STAMBP* mutations. Our study is the first to provide evidence that *STAMBP* plays an important role in neocortical development by maintaining neural stem cell proliferation. *STAMBP* deficient organoids showed a reduction in organoid size due to disruption of the proliferation of NSCs. There was no significant difference in apoptosis between *STAMBP* KO hCOs and controls during brain development. Notably, the microcephaly phenotype was not recapitulated in *Stambp* null mice. Ishii et al. (2001) reported that *Stambp*-deficient mice were morphologically indistinguishable from their littermates at birth and assumed that apoptotic activation was the major pathogenic mechanism of *STAMBP* mutation. Although mouse models can capture some clinical features of human conditions, human organoids provide unique functions in the study of human diseases and complement animal models. The pathophysiological mechanism that associates *STAMBP* mutations with microcephaly needs to be studied using multiple suitable models.

In conclusion, we present a Chinese patient with *STAMBP* mutation, and a novel pathogenic compound heterozygosity of the *STAMBP* gene broadening the spectrum of phenotypic presentations associated with *STAMBP* mutations. The results of the present study may improve our understanding of STAMBP function in human cortical development.

## Data availability statement

The original contributions presented in this study are included in the article/Supplementary material, further inquiries can be directed to the corresponding authors.

## Ethics statement

The studies involving human participants were reviewed and approved by Ethics Committee of Children’s Hospital affiliated to Fudan University (permit no. 2016-131). Written informed consent to participate in this study was provided by the participants or their legal guardian/next of kin. Written informed consent was obtained from the minor(s)’ legal guardian/next of kin for the publication of any potentially identifiable images or data included in this article.

## Author contributions

HL, WF, and XX: conceptualization and supervision. WF, MH, ZH, YX, and BC: organoid methodology. HL, DL, QX, YW, and JD: clinical data collection and analysis. MH, HL, WF, and XX: investigation. MZ: data curation. MH and HL: writing – original draft. WF and XX: writing – review and editing. All authors contributed to the article and approved the submitted version.

## Footnotes

1
http://genetics.bwh.harvard.edu/pph2/

2
http://provean.jcvi.org/index.php

3
https://pubmed.ncbi.nlm.nih.gov/

4
https://www.cnki.net/

5
https://new.wanfangdata.com.cn/index.html

6
https://www.ncbi.nlm.nih.gov/clinvar/

7
https://www.benchling.com/

## Supplementary material

Supplementary Figure 1 *STAMBP* deletion did not affect the pluripotency of hESCs. Immunostaining and RT-PCR were done to confirm the pluripotency of H9 human embryonic stem cells (hESCs) before brain organoid generation. **(A)** Immunostaining analysis showed no significant changes in the expression of pluripotent markers NANOG and SOX2 between *STAMBP* KO hESCs and control. Scale bar, 100 μm. **(B)** RT-PCR showed that *STAMBP* KO hESCs have no significant changes in the expression of pluripotent markers SOX2 and REX1 compared to control. Error bars represent SEM of three independent experiments containing numbers of organoids as indicated; ns *p* > 0.05 (Student’s *t*-test).

Supplementary Figure 2 The variants of patients were successfully inserted. **(A)** The protein structure shows the position of Gly307 and Thr313. **(B)** Sanger sequencing showed the variants c.920G>A (p.Gly307Glu) and c.938C>T (p.Thr313Ile) were inserted.
