# Supplementary figures and images for: Novel compound heterozygous mutation in STAMBP causes a neurodevelopmental disorder by disrupting cortical proliferation
Source: Front Neurosci. 2022 Aug 10;16:963813. doi: 10.3389/fnins.2022.963813 (PMC9399766; doi:10.3389/fnins.2022.963813)

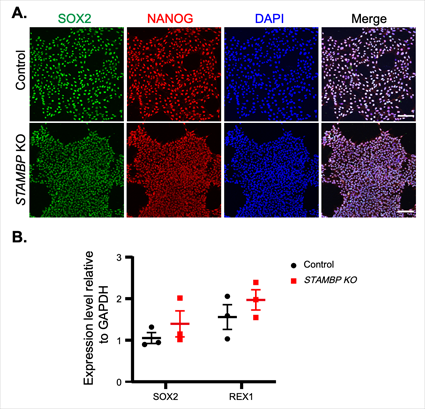

Supplement: Supplementary Figure 1 — STAMBP deletion did not affect the pluripotency of hESCs. Immunostaining and RT-PCR were done to confirm the pluripotency of H9 human embryonic stem cells (hESCs) before brain organoid generation. (A) Immunostaining analysis showed no significant changes in the expression of pluripotent markers NANOG and SOX2 between STAMBP KO hESCs and control. Scale bar, 100 μm. (B) RT-PCR showed that STAMBP KO hESCs have no significant changes in the expression of pluripotent markers SOX2 and REX1 compared to control. Error bars represent SEM of three independent experiments containing numbers of organoids as indicated; ns p > 0.05 (Student’s t-test). [file Image_1.TIFF]

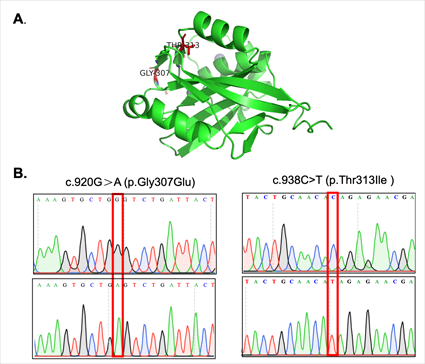

Supplement: Supplementary Figure 2 — The variants of patients were successfully inserted. (A) The protein structure shows the position of Gly307 and Thr313. (B) Sanger sequencing showed the variants c.920G>A (p.Gly307Glu) and c.938C>T (p.Thr313Ile) were inserted. [file Image_2.TIFF]
