# Supplementary material for: Novel compound heterozygous mutation in STAMBP causes a neurodevelopmental disorder by disrupting cortical proliferation
Source: Front Neurosci. 2022 Aug 10;16:963813. doi: 10.3389/fnins.2022.963813 (PMC9399766; doi:10.3389/fnins.2022.963813)
Supplement: Supplementary file 3 [file Table_1.docx]

**Supplementary table1. Summary of the patient and 20 previous reported STAMBP mutation cases.**

| patient | 1 | 2 | 3 | 4 | 5 | 6 | 7 | 8 | 9 | 10 | 11 |
| --- | --- | --- | --- | --- | --- | --- | --- | --- | --- | --- | --- |
| Reference | This case | McDonell *et al* 2013 | | | | | | | | | |
| Validated mutations (protein alteration(cDNA)) | p.C282Wfs*11(c.843_844del)  p.G307E(c.920G > A) | p.Glu42Gly (c.125A>G)    p.Arg178* (c.532C>T) | p.Glu42Gly (c.125A>G)  p.Arg178* (c.532C>T) | p.Arg38Cys (c.112C>T)  c.279+5G>T | p.Phe100Tyr (c.299T>A)  p.Arg424* (c.1270C>T) | p.Arg424*  (c.1270C>T)  p.Arg424*  (c.1270C>T) | c.1005+358A>G  c.1005+358A>G | p.Lys378Asnfs*2 (c.1134_1138  delACTAA)  p.Thr313Ile (c.938C>T) | p.Arg38Cys (c.112C>T)  c.203+5G>A | p.Arg38Cys (c.112C>T)  p.Ile138Serfs*12 (c.411del C) | p.Tyr63Cys (c.188A>G)  p.Arg14Pro (c.41G>C) |
| Gender | F | F | M | M | M | M | F | M | F | F | M |
| Age | 3 years 11 months | 2 years | 9 months | 12 months | 2 years | 22 days | 5 years 4 months | 2 months | 28 months | 8 months | 15 months |
| Ethnicity | Chinese | African-American | African-American | European | European | European | European | European | European | Polynesian | European |
| GA, BW (wees, SD or g) | Term, 2800 | 39, -1.5 | 39, -1.5 | 36^+5^, -1.5 | 36, -2 | 37, -2 | Term, +1.8 | 36, -1.5 | 37^+2^, -4 | 37^+6^, -1.5 | 35, -1.5 |
| **Symptoms and signs** |  | | | | | | | | | | |
| Short stature | **+** | **-** | **+** | **+** | **+** | **+** | **-** | **+** | **+** | **-** | **+** |
| Microcephaly | **+** | **+** | **+** | **+** | **+** | **+** | **+** | **+** | **+** | **+** | **+** |
| Capillary malformations | **-** | **+** | **+** | **+** | **+** | **+** | **+** | **+** | **+** | **+** | **+** |
| Dysmorphic appearance | **+^1,5^** | **+^3^** | **+^3^** | **-** | **+^3^** | **+^3^** | **+^3^** | **+^3^** | **+^3^** | **+^3^** | **+^3^** |
| Intractable epilepsy (age of onset) | **+**(12 months) | **+**(NA) | **+**(NA) | **+**(NA) | **+**(NA) | **+**(NA) | **+**(NA) | **+**(NA) | **+**(NA) | **+**(NA) | **+**(NA) |
| Infantile spasms | - | + | - | + | - | - | - | - | NA | + | + |
| Myoclonus | - | - | + | + | + | - | - | + | + | NA | + |
| Developmental delay | + | + | + | + | + | + | + | + | + | + | + |
| Autism-like behavior | + | NA | NA | NA | NA | NA | NA | NA | NA | NA | NA |
| Spastic quadriparesis | - | + | + | + | + | + | - | - | + | + | + |
| Optic atrophy | - | + | + | + | + | + | NA | NA | + | - | - |
| Dyskinesia | - | NA | NA | NA | NA | NA | NA | NA | NA | + | NA |
| **Auxiliary examination** |  | | | | | | | | | | |
| Neuroimaging features | **-** | **+^c-e^** | **+^c-e^** | **+^c-e^** | **+^c,d^** | **+^c-e^** | **-** | **+^c-e^** | **+^c-e^** | **+^c-e^** | **+^c,d^** |
| EEG anomalies | **+** | Presume + | Presume + | Presume + | Presume + | Presume + | Presume + | Presume + | Presume + | Presume + | Presume + |

| patient | 12 | 13 | 14 | 15 | 16 | 17 | 18 | 19 | 20 | 21 | Total |
| --- | --- | --- | --- | --- | --- | --- | --- | --- | --- | --- | --- |
| Reference | Pavlovic *et al* 2014 | | Faqeih *et al* 2015 | | Naseer *et al* 2016 | | Hori *et al* 2018 | Demikova *et al* 2018 | WU *et al* 2019 | LM *et al* 2019 |  |
| Validated mutations (protein alteration(cDNA)) | (IVS 9-6T>G)  (IVS 9-6T>G) | (IVS 9-6T>G)  (IVS 9-6T>G) | c.1119-6T>G  c.1119-6T>G | c.1119-6T>G  c.1119-6T>G | p.Lys303Arg(c.908A>G)  p.Lys303Arg(c.908A >G) | p.Lys303Arg(c.908A>G)  p.Lys303Arg(c.908A >G) | p.Ser236Phe(c.707C>T)  p.Ser236Phe(c.707C>T) | c.273delA  c.204-5 C > G | p.Q323R(c.968A>G)  c.1119-1G>T | p.Tyr63Cys (c.188A>G)  p.Tyr63Cys (c.188A>G) |  |
| Gender | M | M | M | M | M | M | M | F | M | M | 6F/15M |
| Age | 7 years | 12 years | 8 years 6 months | 5 years | NA | NA | 2 years | 6 months | 1 year 3 months | 1 year and 6 months |  |
| Ethnicity | Saudi | | Arabic | | Egyptian | | Japanese | Russia | Chinese | Russia | 9 |
| GA, BW (wees, SD or g) | 39, 5200 | 39, 4200 | Term, 2600 | NA, 2800 | NA | NA | 37, 2680 | 30, 2250 | 40^+5^, 3600 | Term, 2280 |  |
| **Symptoms and signs** |  | | | | | | | | | | |
| Short stature | + | + | + | + | NA | NA | + | + | + | + | 16(76.2%) |
| Microcephaly | **+** | **+** | **+** | **+** | **+** | **+** | **+** | **+** | **+** | **+** | 21(100%) |
| Capillary malformations | **+** | **+** | **+** | **+** | **+** | **+** | **+** | **+** | **+** | **+** | 20(95.2%) |
| Dysmorphic appearance | +^3,5^ | +^1,3,5^ | +^1,2^ | +^1,2,3^ | +^1,2,3^ | +^1,2,3^ | +^1,2,4^ | +^1,4^ | +^1,4^ | +^1,3,4^ | 20(95.2%) |
| Intractable epilepsy (age of onset) | +(7 months) | +(7 months) | +(7 months) | +(7 months) | +(infancy) | +(infancy) | +(7 months) | +(2 months) | +(3 months) | +(1 month) | 21(100%) |
| Infantile spasms | NA | NA | NA | NA | NA | NA | + | + | + | + | 8(38.1%) |
| Myoclonus | NA | NA | NA | NA | NA | NA | NA | + | + | + | 9(42.9%) |
| Developmental delay | + | + | + | + | + | + | + | + | + | + | 21(100%) |
| Autism-like behavior | + | + | NA | NA | NA | NA | NA | NA | NA | NA | 3(14.3%) |
| Spastic quadriparesis | NA | NA | + | + | NA | NA | - | + | + | + | 13(61.9%) |
| Optic atrophy | + | + | + | + | + | + | - | NA | NA | + | 13(61.9%) |
| Dyskinesia | + | + | NA | NA | NA | NA | NA | NA | + | NA | 4(19.0%) |
| **Auxiliary examination** |  | | | | | | | | | | |
| Neuroimaging features | +^c,d^ | +^c,d^ | +^d^ | +^d^ | +^c,d^ | +^c,d^ | +^d^ | +^d^ | +^d^ | +^c,d^ | 19(90.5%) |
| EEG anomalies | + | + | + | + | + | + | + | + | + | + | 21(100%) |

NA: not available

GA: gestation age, BW: birth weight

Dysmorphic appearance^a^: ^1^widely spaced eyes; ^2^long palpebral fissures; ^3^underdevelopmed distal phalanges; ^4^downturned mouth; ^5^malformed ear or low-set ear

Neuroimaging features^b^: ^c^simplified gyral pattern; ^d^cerebral atrophy/ increased extra-space; ^e^hippocampal hypoplasia.

Presume +: These cases had intractable epilepsy but without detailed EEG data.

EEG: electrophalography
