# Supplementary material for: Novel compound heterozygous mutation in STAMBP causes a neurodevelopmental disorder by disrupting cortical proliferation
Source: Front Neurosci. 2022 Aug 10;16:963813. doi: 10.3389/fnins.2022.963813 (PMC9399766; doi:10.3389/fnins.2022.963813)
Supplement: Supplementary file 4 [file Table_2.docx]

**Supplementary Table2. Name, source and catalogue of antibodies.**

| Antibody Name | Source | Catalogue number |
| --- | --- | --- |
| STAMBP | Santa Cruz Biotechnolgy | sc-271641 |
| GAPDH | Cell Signaling Technology | 97166 |
| FLAG | shanghai genomics | GNI4110 |
| SOX2 | R&D Systems | MAB2018 |
| NANOG | Servicebio | GB11331 |
| PAX6 | MBL | PD022 |
| DCX | Cell Signaling Technology | 4604 |
| NeuN | Cell Signaling Technology | 12943 |
| Ki67 | Servicebio | GB111499 |
| CC3 | Cell Signaling Technology | 9661 |
| PH3 | Cell Signaling Technology | 9701 |
