# Supplementary material for: Novel compound heterozygous mutation in STAMBP causes a neurodevelopmental disorder by disrupting cortical proliferation
Source: Front Neurosci. 2022 Aug 10;16:963813. doi: 10.3389/fnins.2022.963813 (PMC9399766; doi:10.3389/fnins.2022.963813)
Supplement: Supplementary file 5 [file Table_3.docx]

**Supplementary Table3. Name of primer of RT-qPCR.**

| Name | Forward (5’-3’) | Reverse (5’-3’) |
| --- | --- | --- |
| SOX2 | CCCACCTACAGCATGTCCTACTC | TGGAGTGGGAGGAAGAGGTAAC |
| REX1 | TGGACACGTCTGTGCTCTTC | GTCTTGGCGTCTTCTCGAAC |
| STAMBP | CCTCATCACACTGGGCTGGATT | GGCTACTGACTCTGGCAACATC |
