# Supplementary material for: Novel compound heterozygous mutation in STAMBP causes a neurodevelopmental disorder by disrupting cortical proliferation
Source: Front Neurosci. 2022 Aug 10;16:963813. doi: 10.3389/fnins.2022.963813 (PMC9399766; doi:10.3389/fnins.2022.963813)
Supplement: Supplementary file 6 [file Table_4.docx]

**Supplementary Table4. Name, source and catalogue of chemicals and recombinant proteins.**

| Name | Source | Catalogue number |
| --- | --- | --- |
| mTeSR Plus | Stemcell | 100-0274 |
| Accutase | Sigma | A6964 |
| FBS | Thermo Fisher Scientific | 10270-106 |
| KnockOut Serum Replacement | Thermo Fisher Scientific | A3181502 |
| DMEM/F-12 | Thermo Fisher Scientific | C11330500BT |
| Neurobasal Medium | Thermo Fisher Scientific | 21103049 |
| MEM-NEAA | Life Technologies | 11140050 |
| GlutaMAX supplement | Thermo Fisher Scientific | 35050061 |
| β-Mercaptoethanol | Sigma | M7522 |
| LDN-193189 | Sigma | SML0559 |
| SB431542 | Selleck | S1067 |
| XAV939 | Sigma | X3004 |
| Y-27632 | Stem Cell Technologies | 72304 |
| B-27 Supplement | Thermo Fisher Scientific | 17504044 |
| B-27 Supplement without VitaminA | Thermo Fisher Scientific | 12587010 |
| N2 supplement | Thermo Fisher Scientific | 17502048 |
